# Exogenous Hydrogen Sulfide Alleviates Low Temperature and Fluctuating-Light-Induced Photoinhibition of Photosystem I in *Morus alba* Through Enhanced Energy Dissipation and Antioxidant Defense

**DOI:** 10.3390/biology14111582

**Published:** 2025-11-12

**Authors:** Xiaowei Wei, Ju Zhang, Mingyue Sun, Nan Xu

**Affiliations:** 1 Jilin Provincial Key Laboratory for Plant Resources Science and Green Production, Jilin Normal University, Siping 136000, China; weixiaowei@jlnu.edu.cn (X.W.);; 2 Key Laboratory of Heilongjiang Province for Cold-Regions Wetlands Ecology and Environment Research, Harbin University, Harbin 150086, China

**Keywords:** *Morus alba*, low temperature, fluctuating light, hydrogen sulfide (H_2_S), photosystem I (PSI), photosystem II (PSII), non-photochemical quenching (NPQ), antioxidant enzymes

## Abstract

Low temperature combined with fluctuating irradiance frequently co-occurs and suppresses photosynthesis, with irreversible injury to photosystem I (PSI) recognized as a key constraint on growth and yield. To test whether exogenous hydrogen sulfide (H_2_S) mitigates this “cold–fluctuating light” stress in mulberry, we established six treatment combinations (room temperature controls, sodium hydrosulfide, and hypotaurine, each with or without low temperature plus fluctuating light). We quantified PSI/PSII photochemical properties, gas exchange, reactive oxygen species (ROS), and antioxidant enzyme activities. Under cold with fluctuating light, PSI was strongly inhibited: *Y*_NA_ increased, whereas *Y*_I_ and Δ*I*/*I*_o_ decreased, and the P700 re-reduction half-time (t½) was prolonged (ANOVA, Tukey’s HSD, *p* < 0.05), indicating pronounced acceptor-side over-reduction and impaired electron transport. PSII performance also declined (lower *F*_v_/*F*_m_ and *PI*_ABS_, higher Δ*V*_J_; *p* < 0.05). NaHS pretreatment significantly alleviated these effects relative to the stressed control: PSI/PSII metrics partly recovered, net photosynthetic rate (*P*_n_) and water-use efficiency (WUE) increased, H_2_O_2_ and MDA decreased, and SOD/POD/CAT activities rose (*p* < 0.05). Notably, NPQ_high_ correlated negatively with *Y*_NA_ (Pearson r < 0, *p* < 0.001), consistent with the notion that enhanced energy dissipation relieves PSI acceptor-side limitation. We propose that exogenous H_2_S stabilizes electron transport and supports carbon assimilation via a dual strategy—faster engagement of energy dissipation and activation of antioxidant defenses—highlighting its potential utility for managing stress in fruit crops under erratic early-season weather.

## Simple Summary

Sudden cold snaps, often accompanied by rapid fluctuations in sunlight, disrupt leaf light-harvesting mechanisms. In mulberry trees, this combination weakens the photosynthetic apparatus, decreases photosynthetic and water-use efficiency, and increases oxidative stress. To investigate potential mitigation, we tested the effect of a small, safe dose of hydrogen sulfide (H_2_S), a naturally occurring gas in plants. Young mulberry plants were exposed to low temperatures and fluctuating light, with or without a one-time leaf spray that gradually releases H_2_S. Under stress, photosynthesis declined, and oxidant levels increased. H_2_S treatment, however, enhanced photosynthesis and water-use efficiency, reduced oxidant accumulation, and activated endogenous protective enzymes. Two mechanisms were observed: excess light energy was dissipated as heat, and antioxidant defenses were enhanced. These effects stabilized energy flow and improved carbon utilization under stress. The results indicate that low-dose H_2_S could serve as a practical strategy for mitigating the impact of erratic spring weather on fruit tree yield.

## 1. Introduction

Low temperature and fluctuating irradiance rank among the predominant abiotic constraints on both crop productivity and ecosystem stability [1]. In the field, these factors often coincide—early-spring cold spells under clear skies are a typical example—and the combined insult to the photosynthetic apparatus exceeds that of either stress alone. Mulberry (*Morus alba* L.), while economically important, is notably sensitive to chilling; under such composite stress, its photosynthetic capacity is curtailed, with direct consequences for growth and yield [2]. Accordingly, resolving the mechanism of photoinhibition under this dual stress and identifying actionable mitigation routes is of clear scientific and agronomic interest.

Although PSII has long been regarded as the principal target of photoinhibition, a growing body of work shows that PSI is more vulnerable when low temperature intersects with strong—particularly fluctuating—light, becoming an earlier and more critical lesion along the electron-transfer chain [3,4]. In this “cold–light shock” context, Calvin-cycle carbon assimilation is slowed by temperature, whereas light reactions during high-light phases continue to drive substantial electron flux [5]. The resultant disequilibrium between supply and consumption congests the PSI acceptor side, producing over-reduction, stimulating reactive oxygen species (ROS) formation, and ultimately inflicting irreversible damage on the PSI complex [6]. Relieving acceptor-side over-reduction at PSI, therefore, emerges as the central problem in protecting plants from this composite stress.

Hydrogen sulfide (H_2_S) has been recognized as a gaseous signal widely engaged in plant acclimation to abiotic challenges [7]. Low-dose exogenous H_2_S enhances stress tolerance, mitigating cold-induced oxidative injury via activation of antioxidant machinery [8,9] and improving cold hardiness through regulation of key transcriptional modules [10,11]. Together, these observations position H_2_S as a promising modulator of chilling tolerance.

Even so, how H_2_S functions under the low temperature–fluctuating-light regime—where PSI acceptor-side over-reduction is the defining feature—remains unresolved. Whether H_2_S can precisely alleviate that over-reduction, and by which photoprotective route—augmentation of non-photochemical quenching (NPQ) or activation of antioxidant defenses—is a matter for experimental clarification. To this end, mulberry seedlings were employed to establish a “cold–light shock” model and to evaluate, in a systems manner, the protective effects of exogenous H_2_S on the photosynthetic apparatus, with emphasis on PSI regulation and the underlying mechanisms of energy partitioning and antioxidant defense. We asked whether a low, single foliar dose of an H_2_S donor could stabilize PSI under cold–fluctuating light by enhancing energy dissipation and antioxidant capacity. From an agronomic perspective, such a simple pretreatment timed to erratic early-spring weather could help fruit crops maintain photosynthesis and water-use efficiency.

## 2. Materials and Methods

### 2.1. Plant Material and Growth Conditions

One-year-old seed-propagated mulberry (*Morus alba* L., cv. Tailai) seedlings of uniform vigor were selected. Plants were grown in a peat/perlite substrate (3:1, *v*/*v*) and acclimated for 7 d in a controlled-environment chamber (day/night 25/18 °C; 12 h/12 h light/dark; PPFD 200 μmol m^−2^s^−1^, relative humidity 70–80%).

### 2.2. Experimental Design and Treatments

The experiment followed a split-plot arrangement. The main factor was the environmental regime. Seedlings either grew at room temperature (25/18 °C) under constant irradiance of 200 μmol m^−2^s^−1^, or they were subjected to a combined stress of low temperature (4 °C) and fluctuating light. The fluctuating-light program alternated between 2 min of low light (50 μmol m^−2^s^−1^) and 2 min of high light (600 μmol m^−2^s^−1^) for a total of 2 h each morning. Light shifts were nearly instantaneous (<1 s), and the LED panels (broad spectrum 400–700 nm, peaks ~450 and 660 nm) switched between low and high PPFD within <1 s; intensities were verified at the leaf level with a calibrated quantum sensor prior to each run.

Within each environment, three chemical treatments were applied: CK (buffer control), NaHS 100 μM (H_2_S donor), and hypotaurine (HT) 200 μM (H_2_S scavenger). Solutions were prepared in 10 mM MES (pH 6.0) with 0.05% (*v*/*v*) Tween-20 and sprayed to run-off on both leaf surfaces 24 h before the first stress treatment. The 200 μM HT dose follows published plant H_2_S-scavenging protocols and is considered non-injurious; in a brief test on mulberry leaves, it caused no chlorosis or electrolyte leakage within 72 h(Table 1).

Each treatment combination consisted of five individual plants, which were placed randomly inside the chamber to avoid positional effects. Experimental duration and sampling. Treatments were applied for 3 consecutive days; on each morning, plants underwent the fluctuating-light (FL) program for 2 h (50↔600 μmol m^−2^ s^−1^, 2 min per step). For each treatment, five plants (*n* = 5) were used as biological replicates. Unless otherwise noted, one fully expanded, sun-exposed leaf per plant (the 3rd–4th leaf from the apex) was sampled per assay. Gas exchange and PSI/PSII photochemistry were recorded on day 3 during/after the FL session. ROS (H_2_O_2_), MDA, and antioxidant enzymes (SOD, POD, CAT) were determined from independent leaves per plant harvested immediately after the physiological measurements and snap-frozen in liquid N_2_. Pigment contents (Chl a, Chl b, Chl a + b) were quantified on day 3 [12,13].

### 2.3. Measurements of Photosynthetic Performance

All measurements were taken immediately at 0 h post-treatment and after 2 h recovery on each treatment day. The 2nd–3rd fully expanded, healthy leaves were selected and dark-adapted for 20 min before measurement.

#### 2.3.1. Photosystem I (PSI)

A Dual-PAM-100 (Walz, Nuremberg, Germany) was used to determine *Y*_I_, *Y*_ND_, and *Y*_NA_ following standard saturating-pulse protocols [14,15]. P700 redox changes were monitored as the 820–875 nm transmission difference. After far-red pre-illumination, a saturating pulse (10 000 μmol m^−2^s^−1^, 300 ms) was applied to obtain the maximal oxidizable P700 signal amplitude (Δ*I*/*I*_o_) [14]. The dark re-reduction half-time (t½) after the pulse was recorded as an indicator of cyclic electron flow activity [16].

During PSI yield measurements, actinic light (AL) at 200 μmol m^−2^ s^−1^ (RT regime) or at the instantaneous FL level (50 or 600 μmol m^−2^ s^−1^ during the respective phase) was applied. After far-red (FR) pre-illumination (20 s), a 300 ms saturating pulse (10,000 μmol m^−2^ s^−1^) was delivered to determine *Y*_I_, *Y*_ND_, and *Y*_NA_ following Schreiber & Klughammer (2016) [14]; Δ*I*/*I*_o_ was obtained after dark-adaptation and FR pre-illumination as the maximal P700 oxidation amplitude. The P700 dark re-reduction half-time (t½) was recorded immediately post-pulse in darkness.

#### 2.3.2. Photosystem II (PSII)

A multifunction plant efficiency analyzer (M-PEA; Hansatech Instruments, Norfolk, UK) was used to record OJIP transients under 1 s saturating red light (>3000 μmol m^−2^s^−1^). PSII maximum photochemical efficiency (*F*_v_/*F*_m_) and the performance index on an absorption basis (*PI*_ABS_) were calculated according to the JIP-test framework [17,18]. The relative variable fluorescence at J (Δ*V*_J_ at 2 ms) was used to assess the reduction state of the PSII acceptor side [19]. OJIP-derived indices. ABS/RC (apparent antenna size per active PSII RC) and Sm (normalized total complementary area; proxy for PQ-pool/acceptor capacity) were computed from fast fluorescence transients after dark adaptation using standard equations [17,18,19].

### 2.4. Energy Dissipation and Gas Exchange

Non-photochemical quenching (NPQ) was determined with the Dual-PAM-100 [20]. NPQ was recorded at the midpoint of the low-light phase (NPQ_low_) and at the end of the high-light phase (NPQ_high_) during the fluctuating-light cycle. NPQ_low_ was sampled at the 60th s within each 2 min low-light phase (50 μmol m^−2^ s^−1^), and NPQ_high_ at the 120th s of each 2 min high-light phase (600 μmol m^−2^ s^−1^); the FL program ran for 2 h. This timing captures a quasi-steady level within each phase rather than the initial induction transient. NPQ protocol and calculation. Before the FL run, leaves were dark-adapted for 20 min. A saturating pulse (∼10,000 μmol m^−2^ s^−1^, 300 ms) was then applied to obtain Fm. During the FL cycles, saturating pulses at predefined time points provided Fm′ without further dark adaptation. NPQ was calculated as NPQ = (*F*_m_ − *F*_m_′)/*F*_m_′ (Stern-Volmer equation) [14,20]. Sampling points were fixed as follows: NPQ_low_ at *t* = 60 s of the 50 μmol m^−2^ s^−1^ phase, and NPQ_high_ at *t* = 120 s of the 600 μmol m^−2^ s^−1^ phase [20].

Gas-exchange parameters—including net photosynthetic rate (*P*_n_), stomatal conductance (*G*_s_), transpiration rate (*T*_r_), and intercellular CO_2_ concentration (*C*_i_)—were measured with a LI-6800 (LI-COR, Lincoln, NE, USA). Chamber PPFD and leaf temperature matched the respective treatment conditions; CO_2_ was set to 400 μmol mol^−1^. Instantaneous water-use efficiency was calculated as WUE = *P*_n_/*T*_r_ [21].

### 2.5. Antioxidant Enzyme Activity Assays

Sample preparation. For each plant, ~0.20 g of fully expanded leaf tissue was harvested and kept on ice. Samples were homogenized in 50 mM potassium phosphate buffer (pH 7.0) containing 1 mM EDTA and 1% (*w*/*v*) PVPP, then centrifuged at 12,000× *g*, 15 min, 4 °C. The supernatant was used immediately for enzyme assays. Soluble protein was determined by Coomassie Brilliant Blue G-250/Bradford with BSA as a standard [22].

H_2_O_2_. H_2_O_2_ content was quantified by the titanium sulfate method [8]. Briefly, ~0.10 g FW was homogenized in 1.0 mL of cold 0.1% (*w*/*v*) TCA, centrifuged (12,000× *g*, 10 min, 4 °C), and 200 μL supernatant was mixed with 200 μL 10 mM potassium phosphate buffer (pH 7.0) and 10 μL 1% (*w*/*v*) Ti(SO_4_)_2_. After 30 min at room temperature, the mixture was centrifuged and the absorbance read at 410 nm. H_2_O_2_ was calculated using ε = 0.28 mM^−1^ cm^−1^ and expressed as μmol g^−1^ FW.

MDA: MDA was determined by the TBA procedure. 0.20 mL extract was mixed with 0.80 mL 20% (*w*/*v*) TCA containing 0.5% (*w*/*v*) TBA, heated at 95 °C for 30 min, cooled, and centrifuged. Absorbance at A_532_ and A_600_ was recorded. MDA (nmol g^−1^ FW) was calculated as [6.45 × (A_532_ − A_600_) − 0.56 × A_450_] × (V/W), where V is total extract volume and W is sample fresh weight. Reagents were prepared fresh.

SOD (superoxide dismutase): SOD activity was assayed by inhibition of NBT photoreduction at 560 nm. One unit (U) was defined as the amount of enzyme causing 50% inhibition of NBT reduction under assay conditions. Rates were taken from the initial linear region (typically 20–60 s) and expressed as U mg^−1^ protein min^−1^.

POD (peroxidase): POD activity was measured in the guaiacol/H_2_O_2_ system at 470 nm, using ε_470_ = 26.6 mM^−1^ cm^−1^. Activities were calculated from the linear rate and reported as ΔA_470_·min^−1^·mg^−1^ protein, then, where appropriate, converted to U mg^−1^ protein min^−1^ for consistency.

CAT (catalase): CAT activity was determined by H_2_O_2_ decomposition at 240 nm, using ε_240_ = 39.4 mM^−1^ cm^−1^. Rates were taken from the linear region and expressed as ΔA_240_·min^−1^·mg^−1^ protein (reported as U mg^−1^ protein min^−1^ after conversion).

### 2.6. Photosynthetic Pigments

Chlorophyll a (Chl a) and chlorophyll b (Chl b) were extracted with 80% acetone in darkness until tissue bleaching. After centrifugation, absorbance was read at 663 nm and 646 nm, and pigment contents were calculated using standard equations [2].

### 2.7. Statistical Analysis

Data (*n* = 5 plants per treatment) were checked for normality (Shapiro–Wilk) and homogeneity of variances (Levene). Two-way ANOVA tested the fixed effects of scenario (S) and chemical treatment (T) and their interaction (S × T). When ANOVA was significant, pairwise comparisons were performed using Tukey’s HSD at α = 0.05. Pearson correlations were computed among prespecified traits (e.g., NPQ_high_ and *Y*_NA_). Analyses were performed in SPSS 26, and exact *p* values are reported where informative. Although treatments were arranged with environmental regime as the main factor, replicates were independent plants randomized within chambers; therefore, a fixed-effects two-way ANOVA with Tukey’s HSD provides an appropriate test while controlling familywise error.

## 3. Results

### 3.1. Exogenous H_2_S Alleviates the Inhibition of PSI Under Low Temperature and Fluctuating Light

**Figure 1:**
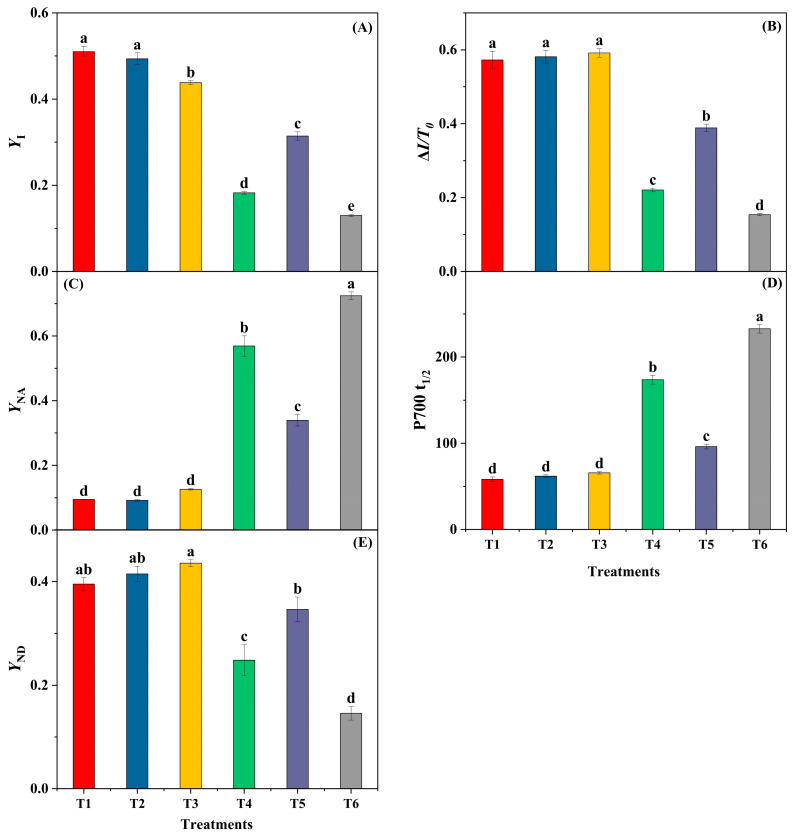
PSI performance under LT + FL. (**A**) *Y*_I_; (**B**) Δ*I*/*I*_o_; (**C**) *Y*_NA_; (**D**) P700 re-reduction (t½); (**E**) *Y*_ND_. Bars = mean ± SE (*n* = 5). Different letters indicate Tukey’s HSD (α = 0.05). (*Y*_I_, Δ*I*/*I*_o_, *Y*_NA_ are dimensionless).

Relative to the room-temperature control (T1), the low-temperature plus fluctuating-light treatment (T4) showed clear inhibition at PSI. The acceptor-side limitation index (*Y*_NA_) was higher, whereas the effective PSI quantum yield (*Y*_I_) and the maximal P700 oxidation amplitude (Δ*I*/*I*_o_) were lower (ANOVA, Tukey’s HSD, *p* < 0.05). In parallel, the P700 dark re-reduction half-time (t½) was prolonged (*p* < 0.05), indicating slower PSI electron turnover and pronounced acceptor-side over-reduction. With NaHS pretreatment (T5), these PSI metrics improved relative to T4—*Y*_NA_ decreased, *Y*_I_ and Δ*I*/*I*_o_ increased, and t½ shortened (all *p* < 0.05). The Hypo group (T6) differed from T4 for some indices but did not exhibit a protective pattern overall (Figure 1).

### 3.2. Exogenous H_2_S Improves PSII Photochemical Performance

**Figure 2:**
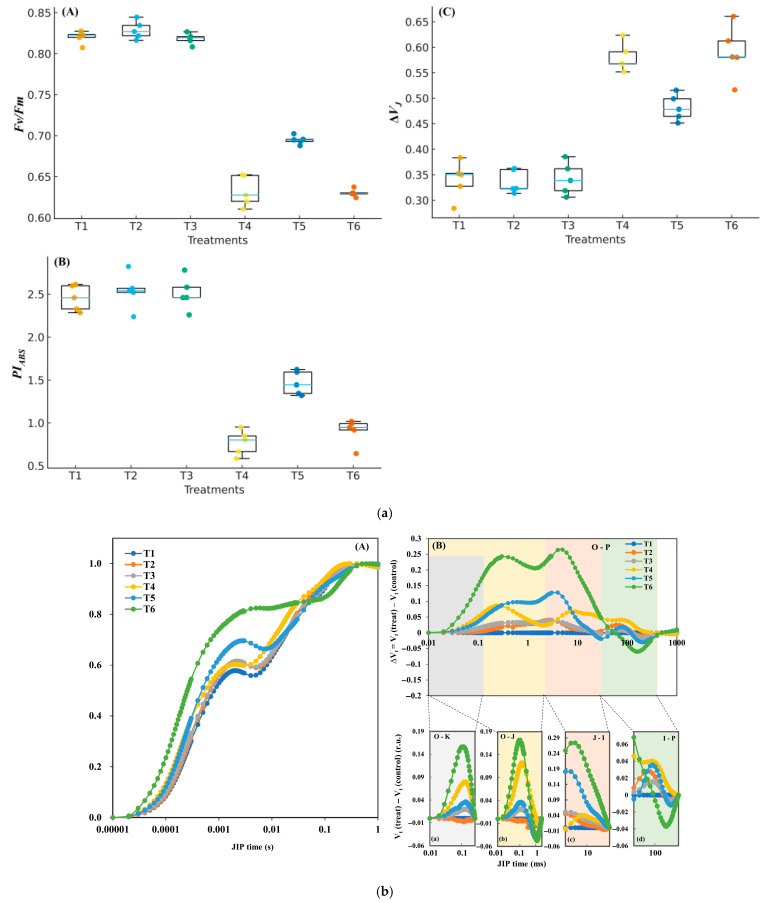
PSII responses and OJIP. (**a**) Box-and-whisker plots of *F*_v_/*F*_m_ (**A**), *PI*_ABS_ (**B**), and Δ*V*_J_ (**C**) (median/IQR/range) with individual points; *n* = 5; different letters indicate Tukey’s HSD (α = 0.05). (**b**) Representative OJIP transients normalized to *F*_0_ under the F_L_ program (2 min at 50 and 2 min at 600 μmol m^−2^ s^−1^; total 2 h). Response of chlorophyll a fluorescence transient (O-J-I-P) curves and differential curves showing differences under different treatments (**A**). Each DC value was calculated as a difference between the values of the relative variable fluorescence [Vt = (F*_t_*– F_0_)/(F*_m_*– F_0_)] recorded of different treatments minus the respective values for T1 treatment, respectively [ΔVt = Vt (treat) − Vt (control)] (**B**). The four characteristic bands are marked with different colors (**a**–**d**), and L band (**a**), K band (**b**), H band (**c**) and G band (**d**) show details in each band. For panels (**a**–**d**) the values for the curves are related to the left scale of Y-axes. Extended indices (ABS/RC, Sm) are in Supplementary Figures S1 and S2. Extended indices corroborated these patterns: ABS/RC increased and Sm decreased under LT + FL relative to RT, and NaHS partially restored both (Tukey’s HSD, α = 0.05; see Supplementary Figures S1 and S2).

Fast chlorophyll fluorescence further indicated PSII impairment under T4. Compared with T1, *F*_v_/*F*_m_ (maximum PSII efficiency) and *PI*_ABS_ (performance index) were lower, whereas Δ*V*_J_ (relative variable fluorescence at the J step) was higher (ANOVA, Tukey’s HSD, *p* < 0.05). Under NaHS (T5), PSII performance partly recovered relative to T4—*PI*_ABS_ increased and Δ*V*_J_ decreased (*p* < 0.05); the improvement in *F*_v_/*F*_m_ was smaller but significant (*p* < 0.05). The Hypo treatment (T6) did not show significant recovery. Representative OJIP transients (Figure 2b) visualize these patterns: under T4 the trace rises steeply at the J step (~2 ms), consistent with stronger reduction on the PSII acceptor side (*Q*_A_); NaHS (T5) dampens this rise, in line with the smaller Δ*V*_J_, whereas Hypo (T6) follows a trajectory similar to the untreated stress condition. ABS/RC increased and Sm decreased under LT + FL relative to RT, and NaHS partially restored both (Tukey’s HSD, α = 0.05; see Supplementary Figures S1 and S2).

### 3.3. H_2_S Protects the Photosynthetic Apparatus by Enhancing Energy Dissipation During High Light

**Figure 3:**
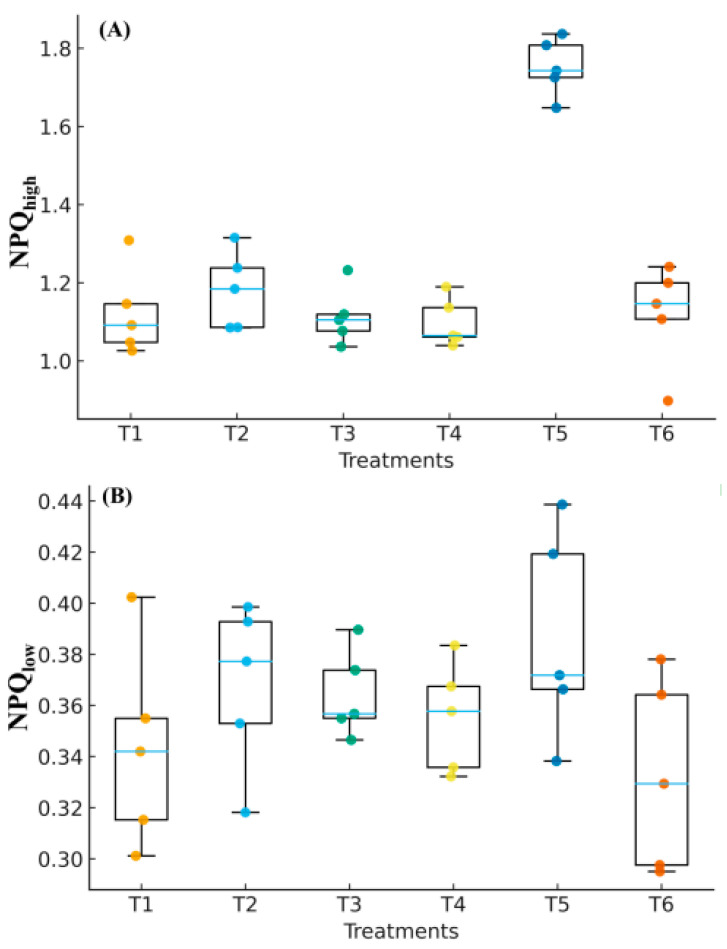
Non-photochemical quenching (NPQ). (**A**) NPQ_low_ at 50 μmol m^−2^ s^−1^, *t* = 60 s; (**B**) NPQ_high_ at 600 μmol m^−2^ s^−1^, *t* = 120 s. Box-and-whisker (median/IQR/range) with individual points; *n* = 5; different letters indicate Tukey’s HSD (α = 0.05). (NPQ is dimensionless.).

Under the fluctuating-light regime, T4 did not show higher NPQ_high_ than T1, and NPQ_low_ likewise showed no change (ANOVA, Tukey’s HSD, *p* > 0.05). With NaHS pretreatment (T5), energy dissipation was significantly enhanced: NPQ_high_ was higher than T4, and NPQ_low_ was also higher (both *p* < 0.05). The Hypo group (T6) did not differ from T4 at NPQ_low_ (*p* > 0.05) but differed at NPQ_high_ (*p* < 0.05), indicating that H_2_S-dependent regulation of NPQ is more prominent under the high-light phase of the FL cycles (Figure 3).

### 3.4. Exogenous H_2_S Improves Gas Exchange and Activates Antioxidant Defenses

Regarding gas exchange, T4 significantly suppressed carbon assimilation relative to T1: net photosynthetic rate (*P*_n_) and water-use efficiency (WUE) were lower (*p* < 0.05). NaHS (T5) partly restored these traits relative to T4—*P*_n_ and WUE were higher (*p* < 0.05)—whereas T6 showed no improvement over T4 (*p* > 0.05).

**Figure 4:**
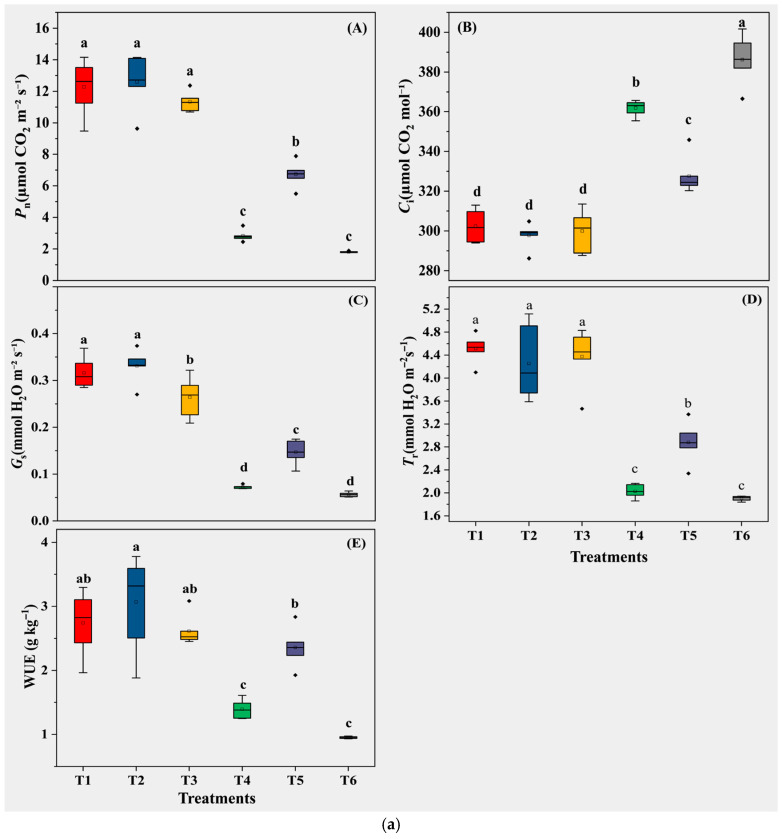

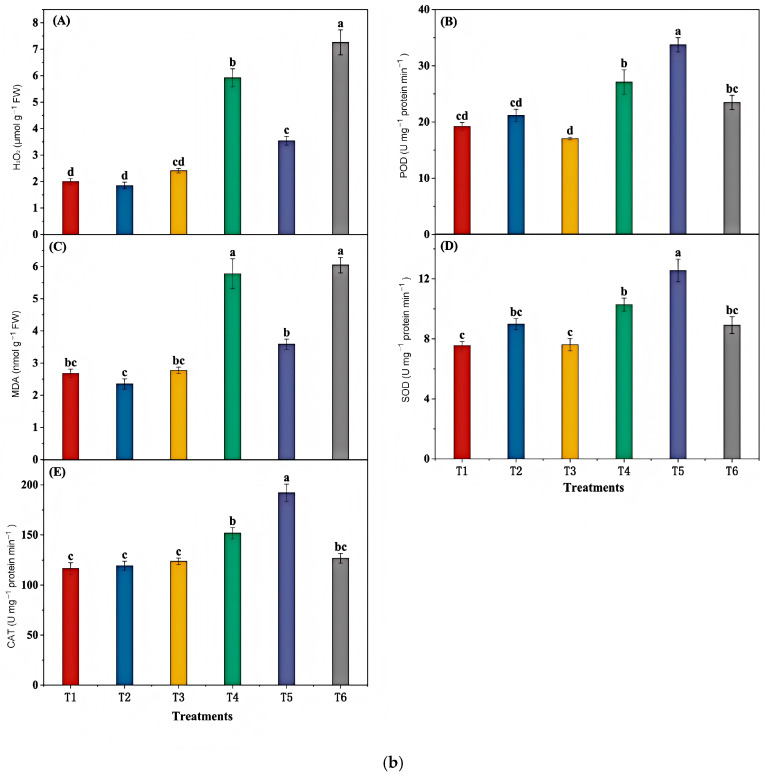
(**a**) Leaf gas exchange and water-use efficiency. (**A**) Net photosynthetic rate (*P*_n_), (**B**) intercellular CO_2_ concentration (*C*_i_), (**C**) stomatal conductance (*G*_s_), (**D**) transpiration rate (*T*_r_), and (**E**) instantaneous water-use efficiency (WUE). Letters denote Tukey’s HSD (α = 0.05). (**b**) Oxidative damage and antioxidant enzymes. (**A**) H_2_O_2_ (μmol g^−1^ FW); (**B**) MDA (nmol g^−1^ FW); (**C**) SOD (U mg^−1^ protein min^−1^); (**D**) POD (U mg^−1^ protein min^−1^); (**E**) CAT (U mg^−1^ protein min^−1^). Bars = mean ± SE (*n* = 5). Different letters indicate Tukey’s HSD (α = 0.05).

For oxidative status, H_2_O_2_ and MDA accumulated in T4 compared with T1 (*p* < 0.05). Relative to T4, T5 showed lower H_2_O_2_ and MDA (*p* < 0.05). In parallel, antioxidant enzyme activities (SOD, POD, and CAT) were higher in T5 than in T4 (*p* < 0.05), while T6 remained similar to T4 (*p* > 0.05) (Figure 4).

### 3.5. Effects of Exogenous H_2_S on Chlorophyll Content and Composition

**Figure 5:**
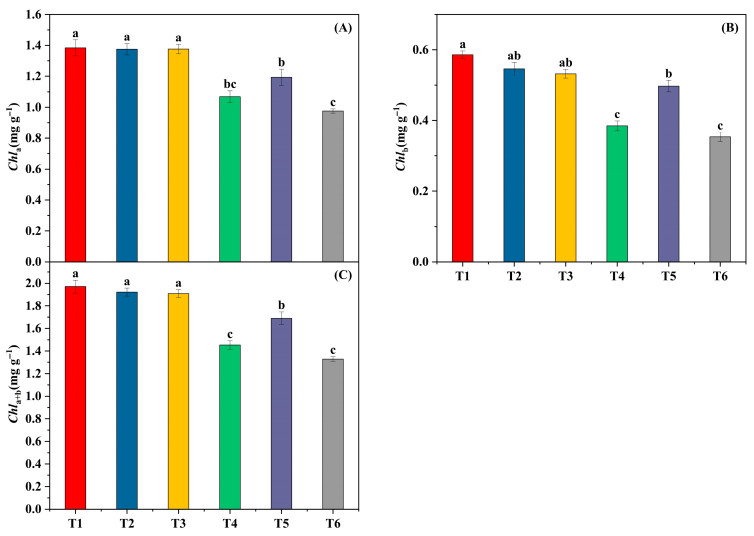
Pigment contents after three days of treatment. (**A**) Chl a; (**B**) Chl b; (**C**) Chl a + b (mg g^−1^). Bars = mean ± SE (*n* = 5). Different letters indicate Tukey’s HSD (α = 0.05).

Relative to the room-temperature control (T1), low temperature with fluctuating light (T4) reduced Chl a and Chl b, and total chlorophyll (Chl a + b) declined accordingly (all *p* < 0.05), indicating a suppression of light-harvesting pigments under cold–light stress. NaHS (T5) significantly increased Chl a and total chlorophyll compared with T4 (*p* < 0.05), with Chl b showing a similar recovery trend, whereas Hypo (T6) remained comparable to T4. These pigment changes parallel the recovery observed for PSI/PSII photochemistry (Figure 5).

### 3.6. Correlation Analysis

**Figure 6:**
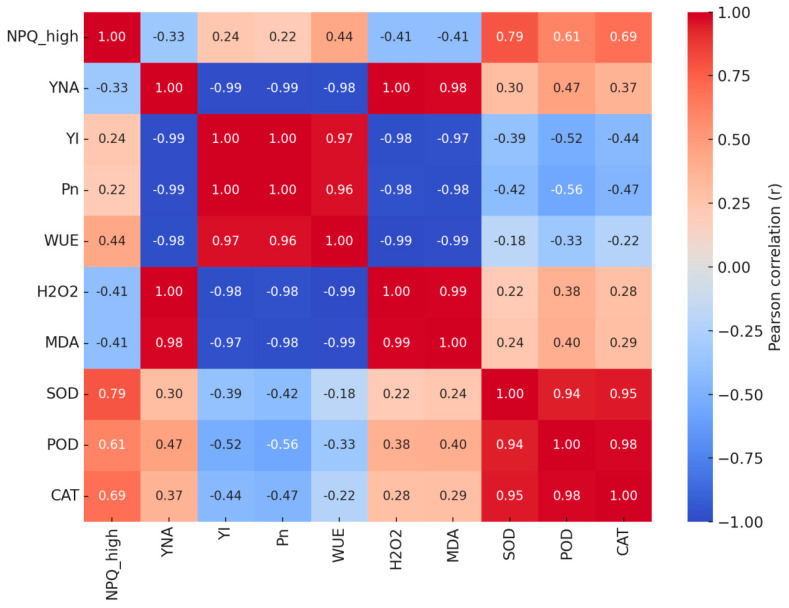
Correlation heatmap among photosynthetic and stress−related traits. The matrix includes PSI performance (*Y*_I_, *Y*_NA_), energy dissipation (NPQ_high_), carbon assimilation (*P*_n_, WUE), oxidative stress markers (H_2_O_2_, MDA), and antioxidant enzymes (SOD, POD, CAT). Pearson’s r values are color−coded, with positive correlations in red and negative in blue. All correlations are based on *n* = 30 (5 reps × 6 treatments).

Pearson’s correlations showed a significant negative relationship between NPQ_high_ and *Y*_NA_ (r = −0.33, *p* < 0.05), indicating that stronger energy dissipation during high light can alleviate PSI over-reduction. *Y*_NA_ was strongly negatively correlated with *Y*_I_, *P*_n_, and WUE (r = −0.98 to −0.99, *p* < 0.001), while *Y*_I_ was almost perfectly positively correlated with *P*_n_ (r ≈ 1.0, *p* < 0.001), suggesting a direct linkage between PSI function and carbon assimilation. H_2_O_2_ and MDA were significantly negatively correlated with *P*_n_ and WUE (r ≈ −0.98 to −0.99, *p* < 0.001). SOD, POD, and CAT were highly positively intercorrelated (r > 0.94, *p* < 0.001), reflecting strong coordination among antioxidant enzymes (Figure 6).

## 4. Discussion

### 4.1. Low-Temperature Fluctuating Light Primarily Impairs PSI Acceptor-Side Function in Mulberry

In natural habitats, plants frequently experience the co-occurrence of low temperature and light fluctuations, and the resulting composite stress is mechanistically more complex than a single factor [1]. Using a combined-stress regime of 4 °C and fluctuating irradiance (50–600 μmol·m^−2^·s^−1^), a pronounced suppression of photosynthetic performance was observed in mulberry, a pattern that may be referred to as “cold–light shock” [2].

Under this regime, the PSI acceptor side emerged as the most vulnerable site. Relative to the room-temperature control, the quantum yield of acceptor-side limitation *Y*_NA_ was higher, whereas *Y*_I_ and Δ*I*/*I*_o_ were lower; the P700 re-reduction half-time (t½) was prolonged (ANOVA, Tukey’s HSD, *p* < 0.05). Together, these shifts identify PSI as the earliest and most sensitive component to sustain damage under cold with fluctuating light, in agreement with earlier reports emphasizing PSI susceptibility under combined chilling and variable irradiance.

Importantly, these findings reinforce the view that PSI, rather than PSII, is the principal bottleneck under such conditions. The dramatic increase in *Y*_NA_ in the stress group (T4) indicates strong over-reduction at the PSI acceptor side, whereas the reduction in PSII maximal efficiency (*F*_v_/*F*_m_) was comparatively modest. This contrast underscores that inhibition of PSI represents the dominant constraint on photosynthetic activity during cold–light stress [3,4].

The mechanistic basis of this selective sensitivity lies in an imbalance between supply and consumption of reducing power. Low temperatures curtail the activity of Calvin cycle enzymes, thereby slowing ATP and NADPH utilization [5]. At the same time, high-light phases continue to drive intense electron input into PSI [6]. The mismatch between demand and supply results in electron congestion at acceptor-side components such as ferredoxin, promoting excessive reactive oxygen species (ROS) accumulation and causing irreversible oxidative injury to the PSI complex [3]. Hence, the present model faithfully recapitulates a composite stress syndrome centered on PSI acceptor-side inhibition and provides a defined target for probing the protective role of H_2_S. We focused on the ecologically frequent co-occurrence of low temperature and fluctuating irradiance, under which PSI acceptor-side over-reduction emerges as an early bottleneck [3,4]. The RT control under constant light provided a stable reference without temperature-imposed metabolic slowdown. While an RT + FL condition could further decompose the role of fluctuation per se, our central question concerned the cold–fluctuating-light composite, for which FL at low temperature is the defining driver of PSI stress; we note this as a limitation and a direction for future work [3,4,5,6,22].

### 4.2. Exogenous H_2_S Alleviates PSI Over-Reduction Primarily by Enhancing NPQ-Mediated Energy Dissipation

With PSI identified as the principal target under “cold–light shock,” the protective route of H_2_S was examined next. Rather than acting on the PSI complex itself, protection was achieved by reallocating excitation and reinforcing non-photochemical quenching (NPQ), the dominant thermal-dissipation pathway for surplus energy [20,23].

Under low temperature with fluctuating irradiance, NPQ_high_ did not rise relative to the room-temperature control (T1), and NPQ_low_ likewise showed no apparent change (T4 vs. T1). Following NaHS pretreatment (T5), however, energy dissipation during the high-light phase was strengthened: NPQ_high_ increased by 64.6% (*p* < 0.05), and NPQ_low_ also exceeded that of the stress control (*p* < 0.05). The Hypo group (T6) differed significantly from T4 at NPQ_high_, but not at NPQ_low_, indicating that H_2_S-dependent regulation is most evident under high-light phases. A significant negative correlation between NPQ_high_ and *Y*_NA_ (r = −0.33, *p* < 0.001) further indicates relief of PSI acceptor-side over-reduction when heat dissipation is enhanced. Consistent with a constrained acceptor side under LT + FL, Sm was lower while ABS/RC was higher; NaHS partly reversed both changes, aligning with the mitigation of PSI acceptor-side limitation reported elsewhere in this study (Figure 1 and Figure 3; Supplementary Figures S1 and S2).

Mechanistically, stronger NPQ is consistent with the faster build-up of the thylakoid proton-motive force (pmf) and activation of the xanthophyll cycle [24,25]. As a signaling molecule, H_2_S may modulate these processes to accelerate NPQ onset and increase its amplitude [7,25,26], thereby dissipating excitation prior to reaction-center engagement, throttling electron inflow from PSII to PSI when carbon assimilation is constrained, restraining the rise of *Y*_NA_, and favoring an oxidized P700 state [27,28]. In addition, it cannot be excluded that H_2_S indirectly promotes PSI protection by stimulating cyclic electron flow (CEF) around PSI, which enhances ΔpH formation and complements NPQ-mediated photoprotection under fluctuating light [26,27]. Collectively, up-regulation of NPQ constitutes a primary line of PSI protection under the cold–light regime.

The absence of higher NPQ in T4 than T1 is explicable because chilling constrains the build-up and partitioning of the proton-motive force, alters thylakoid ion homeostasis, and slows xanthophyll-cycle enzyme kinetics, thereby dampening the qE component despite PSI acceptor-side pressure. NaHS pretreatment likely accelerates pmf formation and facilitates qE engagement specifically during the high-light phase, hence the selective rise in NPQ_high_. Under RT, where induction and qE capacity are already robust, NPQ in T2 need not exceed T1. Conceptually, higher NPQ acts as a “safety valve,” diverting surplus light as heat; this lowers the electron load delivered to PSI, thereby reducing *Y*_NA_, our metric of PSI acceptor-side blockage.

Mechanistic note on qE under LT + FL. At low temperature, counter-ion conductances across the thylakoid (in particular Cl^−^ fluxes and K^+^/H^+^ exchange) are curtailed, which shifts pmf partitioning toward Δψ and slows the buildup of ΔpH at cyt b_6_f. Because qE depends on lumen acidification, a slower ΔpH delays the protonation of PsbS and reduces qE amplitude, providing a parsimonious explanation for the lack of NPQ increase in T4 relative to T1 despite high-light phases. We distinguish the rapid trigger (PsbS protonation) from amplitude tuning by the xanthophyll cycle (VDE/ZE), the latter being temperature-sensitive and modulating the magnitude and persistence of qE [23,24,26].

**Figure 7:**
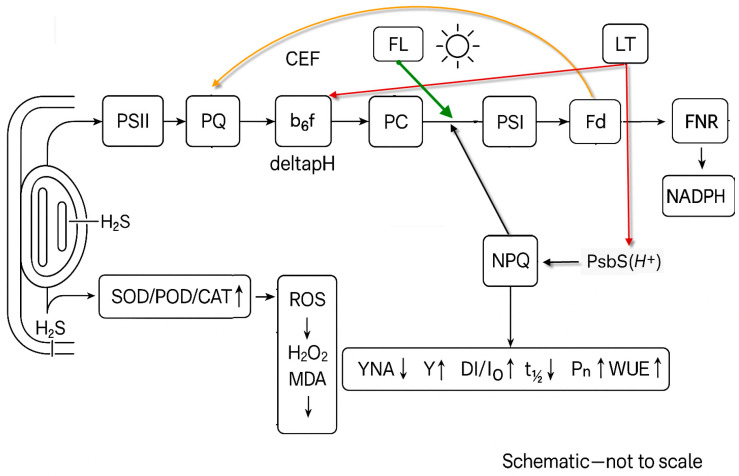
Proposed route by which exogenous H_2_S protects photosynthesis under cold + fluctuating light (LT + FL). Caption: Exogenous H_2_S enters the chloroplast stroma (left). The linear electron transport chain is PSII→plastoquinone (PQ)→cytochrome b6f (b6f)→plastocyanin (PC)→PSI→ferredoxin (Fd)→ferredoxin-NADP^+^ reductase (FNR)→NADPH. Cyclic electron flow (CEF) is depicted as a closed loop via the PQ pool: Fd→PQ→b6f→PC→PSI. deltapH formation is indicated at b6f. A thin arrow from the FL icon to the PC→PSI link denotes the transient increase in load at the PSI input during high-light phases. Protonation of PsbS(H^+^) triggers NPQ (short arrow PsbS(H^+^) → NPQ); a curved feedback arrow from NPQ to the PC→PSI connection indicates reduced excitation/electron inflow to PSI, alleviating acceptor-side limitation. In parallel, H_2_S elevates antioxidant capacity (SOD/POD/CAT up), decreasing ROS and lipid peroxidation (H_2_O_2_/MDA down); thin arrows from this box point to PC and PSI. A small LT tag with thin arrows to b6f and PsbS(H^+^) denotes low-temperature constraints on deltapH build-up and qE triggering, which make the H_2_S-dependent promotion of deltapH/NPQ more evident under LT. Outcomes—*Y*_NA_↓, *Y*_I_ ↑, Δ*I*/*I_0_*,↑, t½↓; Pn↑, WUE↑—match the measured responses. Abbreviations (for the legend): HS, hydrogen sulfide (exogenous H_2_S donor effect); FL, fluctuating light; ETC, electron transport chain; CEF, cyclic electron flow; ΔpH, proton gradient component of pmf; qE, energy-dependent non-photochemical quenching; NPQ, non-photochemical quenching; PsbS(H^+^), protonated PsbS; ROS, reactive oxygen species; H_2_O_2_, hydrogen peroxide; MDA, malondialdehyde; *Y*_NA_, PSI acceptor-side limitation; *Y*_I_, effective PSI quantum yield; Δ*I*/*I_0_*, maximal P700 oxidation amplitude; t½, P700 re-reduction half-time; *P*_n_, net photosynthetic rate; WUE, water-use efficiency; PQ, plastoquinone; b_6_f, cytochrome b_6_f complex; PC, plastocyanin; Fd, ferredoxin; FNR, ferredoxin–NADP^+^ reductase.

How H_2_S could raise NPQ under LT + FL. Exogenous H_2_S likely stabilizes thylakoid redox/ion homeostasis during the high-light phase, thereby facilitating ΔpH formation at cyt b_6_f and enabling faster PsbS engagement, which is consistent with the increase of NPQ_high_ under NaHS and the negative coupling between NPQ_high_ and *Y*_NA_ [7,8,29,30]. We consider this a testable hypothesis that integrates our observations on PSI acceptor-side relief with the known two-tier control of qE (PsbS trigger; xanthophyll-cycle tuning). The revised Figure 7 summarizes this framework, explicitly routing CEF via the PQ pool and indicating ΔpH at b_6_f together with the antioxidant arm that protects PC/PSI. For completeness, CEF is considered along its canonical route via the PQ pool (Fd→PQ→b_6_f→PC→PSI), rather than as a PSI-local loop. In the low-temperature context, restricted counter-ion conductances slow ΔpH build-up at b_6_f, which explains why the H_2_S-dependent promotion of ΔpH and qE/NPQ becomes particularly evident under LT + FL.

Limitations and outlook. While the present study did not assay individual ion fluxes or PsbS protonation directly, the directional NPQ_high_ response to H_2_S and its relationship with *Y*_NA_ support the proposed mechanism. Direct ion-flux and PsbS status measurements under LT + FL ± H_2_S will be informative in future work.

### 4.3. Activation of the Antioxidant System Represents Another Key Pathway Whereby H_2_S Cooperatively Protects the Photosynthetic Apparatus

Even with enhanced NPQ, some reactive oxygen species (ROS) inevitably escape control under composite stress. In the stress control (T4), H_2_O_2_ and MDA levels were markedly elevated relative to T1, signaling strong oxidative pressure with membrane lipid peroxidation. With NaHS pretreatment (T5), enzymatic antioxidant capacity was reinforced: SOD, POD, and CAT activities increased by 26.6%, 24.5%, and 22.1%, respectively, while H_2_O_2_ and MDA declined by 40.2% and 37.9% compared with T4 (all *p* < 0.05). By contrast, the Hypo group (T6) generally resembled T4, showing no protective improvement across these indices. These patterns indicate a reduced oxidative burden through enhanced enzymatic scavenging and preservation of membrane integrity.

Comparable H_2_S-induced mitigation of chilling-related oxidative injury has been reported in other species, supporting antioxidant fortification as a commonly employed arm of H_2_S protection [8,9]. Consequently, PSI is shielded via two coordinated routes: lower ROS formation through stronger NPQ (Section 4.2) and faster ROS removal via activated antioxidant enzymes, acting in concert to stabilize the photosynthetic apparatus.

### 4.4. Recovery of PSI Performance Removes the Bottleneck, Re-Establishes Electron Transport, and Improves Carbon Assimilation

H_2_S-mediated protection initiates at the PSI acceptor side and then propagates through the photosynthetic network. Under cold–light stress, elevated *Y*_NA_ identifies the first bottleneck, restricting electron delivery to ferredoxin. The constraint feeds upstream: the PQ pool remains highly reduced, PSII electron release is curtailed, Δ*V*_J_ increases, and *PI*_ABS_ declines—patterns observed in T4. When NaHS was applied (T5), enhanced NPQ together with stronger antioxidant capacity (Section 4.2 and Section 4.3) relieved acceptor-side load and permitted electron export from PSI. In turn, the PQ pool regained oxidizing potential, PSII back-pressure eased, Δ*V*_J_ decreased, and *PI*_ABS_ rebounded, indicating removal of the key choke point and restoration of chain-wide continuity [8,31]. With electron transport re-established, provision of ATP and NADPH to the Calvin cycle was sustained [32]. Consistently, *P*_n_ and WUE increased in T5 in parallel with photochemical recovery, showing effective conversion of light-reaction stabilization into greater CO_2_ assimilation [21]. The Hypo group (T6) largely mirrored T4, underscoring the necessity of H_2_S for the observed recovery.

By alleviating PSI acceptor-side congestion and restoring chain continuity, NaHS allows more balanced ATP/NADPH provision (via enhanced ΔpH and putative stimulation of cyclic flow), thereby supporting carbon assimilation despite low temperature. Concurrent ROS reduction further preserves Calvin-cycle enzyme function and membrane conductance, translating photochemical stabilization into higher *p*_n and WUE.

Our findings align with broader evidence that relieving PSI stress is central under chilling with variable light. The dual strategy-qE-type NPQ plus antioxidant fortification-offers a practical handle for field interventions (timed pretreatments before forecast cold snaps) and breeding targets (faster NPQ induction and robust ROS-scavenging). Future work should quantify pmf dynamics and cyclic flow under realistic sky-driven light fluctuations and assess field performance across cultivars. A concise mechanistic model summarizing the dual H_2_S protections under cold–fluctuating light is shown in Figure 7.

## 5. Conclusions

Cold–light stress is defined by pronounced over-reduction at the PSI acceptor side, which functions as the dominant constraint on photosynthesis. Exogenous H_2_S counters this constraint via two coordinated arms. First, NPQ during the high-light phase is strengthened (Tukey’s HSD, *p* < 0.05), diverting surplus excitation and lowering the electron load at PSI. Second, antioxidant capacity is reinforced—SOD, POD, and CAT increase by 26.6%, 24.5%, and 22.1%, respectively—accompanied by reductions in H_2_O_2_ (−40.2%) and MDA (−37.9%). The combined effect is the release of the PSI bottleneck, stabilization of electron transport, and a measurable gain in carbon assimilation, evidenced by concurrent recoveries of *P*_n_ and WUE. These outcomes support H_2_S as a credible option for improving tolerance of horticultural crops to cold–light syndromes.

## Figures and Tables

**Table 1:** Experimental processing Settings.

	Treatment
**T1**	RT + CK
**T2**	RT + NaHS
**T3**	RT + Hypo
**T4**	LT + FL + CK
**T5**	LT + FL + NaHS
**T6**	LT + FL + Hypo

## Data Availability

The data that support the findings of this study are available within the article and its Supplementary Mterials.

## Supplementary Materials

The following supporting information can be downloaded at: https://www.mdpi.com/article/10.3390/biology14111582/s1, Figure S1: ABS/RC across treatments (T1–T6); Figure S2: Sm across treatments (T1–T6).

## Abbreviations

RT, room temperature; LT, low temperature (4 °C); FL, fluctuating light (50↔600 μmol m^−2^ s^−1^); PSI/PSII, photosystems I/II; Y(I), effective PSI quantum yield; *Y*_ND_, PSI donor-side limitation; *Y*_NA_, PSI acceptor-side limitation; Δ*I*/*I*_o_, maximal P700 oxidation amplitude; NPQ, non-photochemical quenching; WUE, water-use efficiency; Hypo, hypotaurine (H_2_S scavenger); NaHS, sodium hydrosulfide (H_2_S donor).
