# Supplementary material for: Exogenous Hydrogen Sulfide Alleviates Low Temperature and Fluctuating-Light-Induced Photoinhibition of Photosystem I in Morus alba Through Enhanced Energy Dissipation and Antioxidant Defense
Source: Biology (Basel). 2025 Nov 12;14(11):1582. doi: 10.3390/biology14111582 (PMC12650550; doi:10.3390/biology14111582)
Supplement: Supplementary file 1 [file biology-14-01582-s001.zip › biology-3948835-supplementary.pdf]

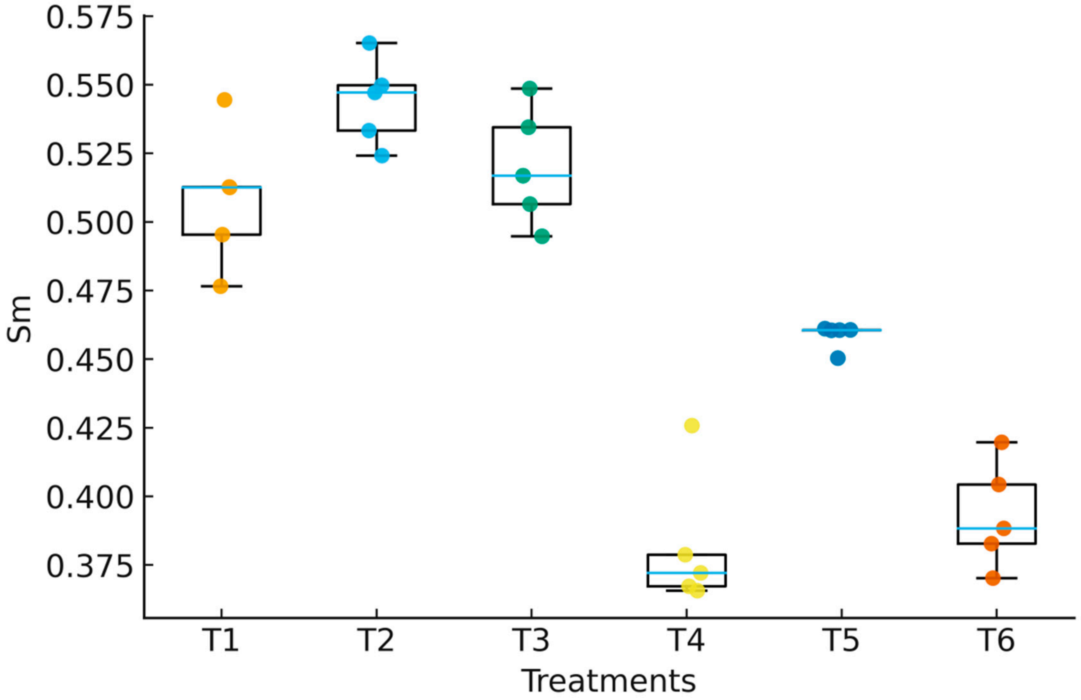

Supplementary Figure S1. ABS/RC across treatments (T1–T6)

Caption:
Box-and-whisker plot of the OJIP-derived index ABS/RC (apparent antenna size per PSII reaction center) for each treatment (T1–T6; see Section 2.2 for treatment definitions). Boxes show the interquartile range (IQR), the central line is the median, whiskers span the data range, and individual plant values ($n = 5$) are overlaid as points. Statistics: Data were tested for normality (Shapiro–Wilk) and homogeneity of variances (Levene), analyzed with a two-way ANOVA (scenario, chemical treatment, and S×T interaction), and pairwise differences were evaluated using Tukey's HSD ($\alpha = 0.05$); different letters indicate significant differences. Interpretation: ABS/RC was higher under LT+FL relative to RT, consistent with partial RC inactivation/energy re-allocation under stress; NaHS (T5) partially reduced the inflation of ABS/RC compared to the stressed control (T4), whereas Hypo (T6) showed no improvement (direction + significance consistent with Results §3.2). Acquisition: Rapid chlorophyll fluorescence (OJIP) after dark adaptation; calculation parameters are detailed in Methods §2.3.2.

Data analysis:
ABS/RC values ($n = 5$ plants per treatment) were analyzed by two-way ANOVA with factors scenario (RT vs LT+FL) and chemical treatment (Control, NaHS, Hypo). Assumptions were checked by Shapiro–Wilk and Levene tests; if needed, data were log-transformed before analysis. When the ANOVA was significant, Tukey's HSD was used for post-hoc pairwise comparisons, and compact letter displays were assigned to groups. Results showed a significant main effect of scenario and a significant S×T interaction ($P < 0.05$), with LT+FL increasing ABS/RC and NaHS partially reversing this effect relative to T4. Analyses were performed in R (aov + TukeyHSD/emmeans) or SPSS (both acceptable; choose one to match your Methods §2.7).

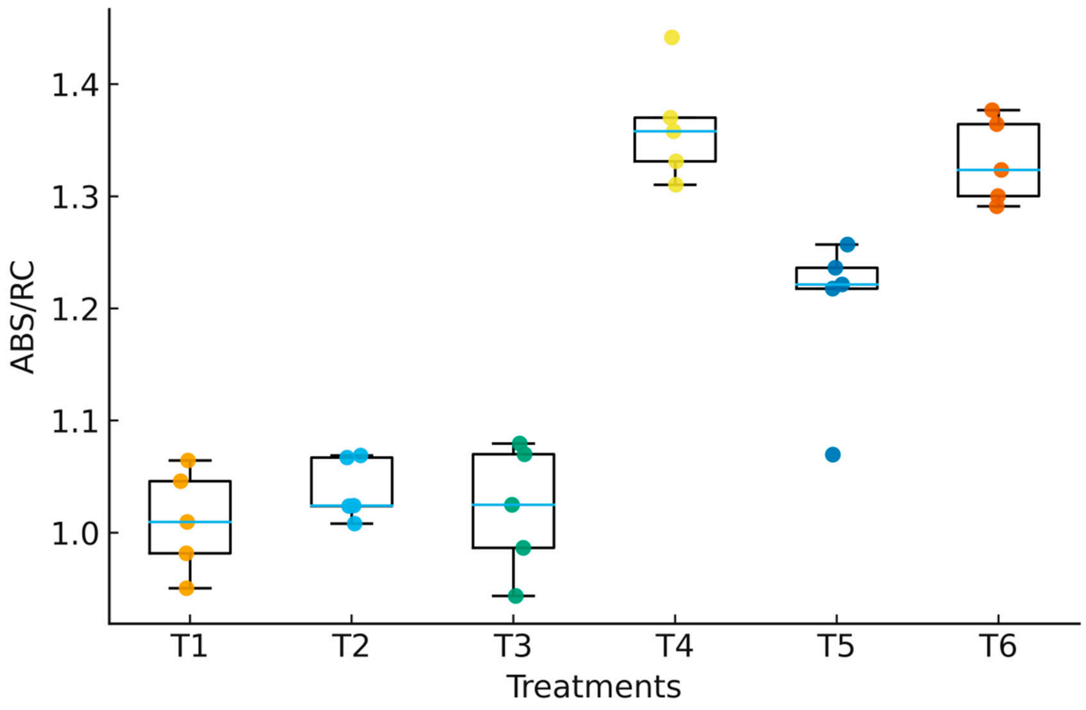

Supplementary Figure S2. Sm across treatments (T1–T6)

Caption :

Box-and-whisker plot of the OJIP-derived index Sm (normalized total complementary area; reflects the PQ-pool/reduction capacity and downstream acceptor availability) for each treatment (T1–T6; definitions in Section 2.2). Boxes show IQR, the central line is the median, whiskers span the data range, and individual plant values ($n = 5$) are overlaid. Statistics: Normality and variance checks as above; two-way ANOVA followed by Tukey's HSD ($\alpha = 0.05$); different letters indicate significant differences. Interpretation: Sm was lower under LT+FL relative to RT, consistent with restricted PQ-pool turnover/acceptor capacity; NaHS (T5) partly restored Sm relative to T4, whereas Hypo (T6) remained similar to the stressed control. Acquisition and calculation follow Methods §2.3.2.

Data analysis :

Sm values ($n = 5$ plants per treatment) were subjected to two-way ANOVA (scenario, chemical treatment, S×T). Normality and variance assumptions were verified (Shapiro–Wilk; Levene) and, if needed, a log transformation was applied. Tukey's HSD provided post-hoc contrasts and letter groupings. The ANOVA indicated significant effects of scenario and a significant S×T interaction ($P < 0.05$), with LT+FL decreasing Sm and NaHS leading to partial recovery compared with T4. Analyses were run in R or SPSS consistent with Methods §2.7.
